# Heterogeneity of adolescent health risk behaviors in rural western China: A latent class analysis

**DOI:** 10.1371/journal.pone.0199286

**Published:** 2018-06-26

**Authors:** Yuanyi Ji, Huanyu Xu, Yu Zhang, Qiaolan Liu

**Affiliations:** West China School of Public Health, Sichuan University, Chengdu, P. R. China; IRCCS E. Medea, ITALY

## Abstract

**Background:**

Adolescent health risk behaviors are a public health priority given their prevalence and their associations with chronic diseases and life quality in adulthood. This study examined the heterogeneity of adolescent health risk behaviors and the associations between demographic characteristics and subgroup membership in rural western China.

**Methods:**

In fall 2015, 2805 students from rural middle schools in Sichuan Province were surveyed using the Health-Related Behavior Questionnaire for Adolescents. Latent class analysis (LCA) was used to identify subgroups of adolescents with distinct patterns of health risk behaviors. Differences in class membership related to selected demographic characteristics were examined using multinomial logistic regression analysis.

**Results:**

A four-class model emerged: (1) high-risk group (n = 108, 4.0%), (2) high-physical-inactivity and suicide-risk group (n = 340, 12.1%), (3) moderate-risk group (n = 897, 32.0%), and (4) low-risk group (n = 1460, 52.1%). The multinomial logistic regression analysis revealed that boys and adolescents with poor parental relationships and high allowances (spending money) were significantly more likely to be in the high-risk group than the low-risk group.

**Conclusions:**

Adolescents in rural western China are a heterogeneous population requiring different tailored and effective interventions.

## Introduction

Adolescent health risk behaviors are an important public health concern worldwide. Health risk behaviors are a serious threat to adolescents’ health and have lasting impacts on life quality in adulthood [1–5]. Additionally, adolescence is a critical time during which many individuals establish independence and adopt lasting health risk behavior patterns that are associated with increased long-term risk for disease [1]. Compared with the economically developed eastern and central regions in China, fewer concerns have been expressed about health risk behaviors in adolescents in western rural areas [6].

Over the past three decades, China’s rapid economic development and unprecedented urbanization have encouraged many rural western parents to migrate to urban centers to pursue employment opportunities [7]. Their children may be left behind in the care of other family members, and some of these children may even care for themselves [8]. Consequently, adolescents suffer from not only a lack of education and health care in rural western areas but also a lack of parental monitoring and companionship during childhood. Evidence is mounting that family instability is associated with a higher likelihood of engaging in health risk behaviors [6,9,10].

Many previous studies have illustrated that the health risk behaviors that emerge during these years are highly correlated [11–14]. For example, adolescents who are at risk for unhealthy diets are also more likely to be physically inactive and have more screen time [15]. However, these traditional variable-centered studies, using methods such as factor analysis and regression analysis, do not provide the key information that a person-centered approach offers regarding the intersection of a wide range of health risk behaviors. Innovative statistical approaches, such as latent class analysis (LCA), enable researchers to examine how these behaviors cluster together among individuals and to gain a broader understanding of health risk behavior patterns [16].

To date, however, there has been scant research examining the combination of individual health risk behaviors and the patterns of these factors in China. This study aimed to investigate the heterogeneity and clustering of health risk behaviors among adolescents in rural western China and the associations between demographic characteristics and health risk behavior subgroups. The findings of the co-occurrence of health risk behaviors in subgroups of adolescents might enable practitioners to improve the effects of interventions and minimize the costs associated with such interventions.

## Materials and methods

### Participants

In total, 2805 rural students from two rural middle schools (total 42 classes) in Zizhong County, Sichuan Province, China, were included in this study. In fall 2015, two middle schools were selected by using the stratified cluster sampling method from 17 rural middle schools located in hilly areas in Zizhong County. In this county, economic development and demographic characteristics, for example, the proportion of migrant workers, are similar to those of the other hilly areas in Sichuan province; thus, these schools offer a certain representativeness with respect to schools in these regions. A total of 2880 questionnaires were sent out, and 2880 were returned, for a response rate of 100%. After excluding the unqualified questionnaires, 2805 effective questionnaires were included in this study. The effective rate was 97.4%. The current cross-sectional research was the first baseline survey of a longitudinal preventive intervention program in rural western China.

### Instruments

#### Demographics

The participants reported their sex, grade level and other demographic characteristics. The students aged 14.7±1.4 years (range 10–17 years) were asked to identify their grade level using a response scale ranging from 7th to 12th grade (junior high school includes grades 7–9, senior high school includes grades 10–12). The number of children in the student’s family, whether they were left-behind children (LBC) and ethnicity, were recorded as dichotomous variables. Parents’ education level (below high school, high school diploma and beyond high school) and parental relationship (good, somewhat poor and poor) were reported in three categories. Academic performance was graded on a five-point scale (good, somewhat good, medium, somewhat poor, poor) in the initial questionnaire. We combined three categories (somewhat good, medium and somewhat poor) into only one category named “moderate”. Therefore, academic performance was also classified into three groups: good, moderate and poor.

#### Health risk behaviors

We used the Health-Related Behavior Questionnaire for Adolescents, a self-administered questionnaire containing questions adapted from the Centers for Disease Control and Prevention’s (CDC’s) Youth Risk Behavior Survey Questionnaire [17] and the Adolescent Health-Related/Risk Behavior Inventory in China [18]. The Cronbach’s α coefficient was 0.81, which meant that this questionnaire had good reliability. Responses to the items about health risk behaviors were used to classify the participants into 2 groups (met the definition or not) for the following 8 behaviors: 1) Unhealthy diet (drinking soft drinks >4 times per day, exhibiting unhealthy weight control behaviors (limiting some food, deliberately vomiting, long-term fasting and taking diet pills) in the past 30 days, eating dessert >2 times per day, eating Western fast food >5 times per week, displaying dietary bias, eating breakfast <2 times per week, drinking milk (yogurt/soybean milk/soy milk) <1 time in the past 7 days); 2) Physical inactivity (engaging in at least 60 minutes of vigorous exercise in <3 of the past 7 days); 3) Unhealthy Internet use (spending >2 hours per day online while in school, spending >4 hours per day online on weekends, and being online overnight in the past 7 days); 4) Accidental injury (having cycling violations or pedestrian violations in the past 30 days or swimming at a location with no safety measures in the past 12 months); 5) Tobacco use (using cigarettes (or hookah, cigars or smokeless tobacco) >1 time during the past 30 days); 6) Binge-drinking (having >5 alcoholic drinks in one sitting in the past 2 weeks); 7) Self-injurious behavior (self-inflicting any intentional injury, including cuts, scalds, bites or scratches in the past 12 months); and 8) Suicide risk (having suicidal thoughts, a suicide plan or suicide attempts in the past 12 months).

### Procedure

Only those students with parental permission were eligible to participate in the study and complete the anonymous surveys (which were administered by trained research assistants) during a class period. Informed written consent was obtained from parents. Furthermore, the research assistants verbally explained the students’ rights as participants in the study, and the students then provided verbal and written consent to participate. Different classes in the same school were surveyed at the same time. The ethics approval of this study was obtained from Medical Ethical Committee of Sichuan University, China (No.20140307).

### Data analysis

First, we analyzed the demographic characteristics and the prevalence of health risk behaviors among the participating rural adolescents. Second, LCA was used to identify the optimal number of latent subgroups and to categorize the participants into different behavior pattern groups according to specific posterior probabilities. Finally, multinomial logistic regression analysis was used to examine the associations between the subgroups and the demographic variables.

LCA, a subtype of structural equation modeling, produces probabilities of item endorsement by class membership. Study participants are grouped by their endorsement patterns, and two informative parameters emerge: (1) the probability of being in a given class for each individual (posterior class probability) and (2) the probability of a response to a certain indicator given a participant’s membership in a latent class (item-class probabilities). Numerous examples of LCA’s potential application, particularly in identifying latent behavioral patterns, have been recently published [19–22]. These study results have demonstrated that LCA is an effective and valid approach that can be used to categorize individuals with similar characteristics.

To our knowledge, selecting an appropriate model with the optimal number of classes is best achieved through the use of fit indices, such as the Akaike information criterion (AIC), the Bayesian information criterion (BIC), and the sample-size-adjusted BIC (aBIC), in which lower values indicate better fit [23]. The Lo-Mendell-Rubin likelihood ratio test (LMR LRT) provides data that help to discern whether a K-class model fits the data better than a (K-1)-class model [24]. A low *P-*value for an LMR LRT indicates that the (K-1)-class model must be rejected in favor of a model with at least K classes. All analyses were conducted using IBM SPSS 20.0 and MPLUS 7.4 with a significance threshold of *α* = 0.05 (two-tailed).

## Results

Table 1 describes the profile of the sample, including the participants’ demographic characteristics, their engagement in the eight health risk behaviors and their backgrounds in terms of individual and family factors. The sample included a high percentage (56.9%) of adolescents who were identified as LBC. The majority of the adolescents’ parents had an educational level below high school. Physical inactivity was the most frequently reported risk behavior: 61.2% of the participants reported engaging in at least 60 minutes of exercise in fewer than 3 of the past 7 days. Of the participants, 47.1% reported experiencing an accidental injury in the past 30 days. Only 6.9% of the participants reported tobacco use in the past 30 days, and 11.7% reported binge- drinking in the past 12 months.

**Table 1:** Demographic and behavioral characteristics of the total sample, 2015 (N = 2805).

Variable	N (%)	Variable	N (%)
**Sex**		**Mother’s education**	
Boys	1328(47.3)	Below high school	2460(87.7)
Girls	1477(52.7)	High school diploma	321(11.4)
**Grade**		Beyond high school	24(0.9)
7th	578(20.6)	**Parental relationship**	
10th	2227(79.4)	Good	2146(76.5)
**Left-behind children**		Somewhat poor	473(16.9)
Yes	1596(56.9)	Poor	186(6.6)
No	1209(43.1)	**Academic performance**	
**Only child**		Good	76(2.7)
Yes	689(24.6)	Moderate	2367(84.4)
No	2116(75.4)	Poor	362(12.9)
**Race/ethnicity**		**Health risk behaviors**	
Han	2785(99.3)	Unhealthy diet	449(16.0)
Others	20(0.7)	Physical inactivity	1717(61.2)
**Monthly allowance**		Unhealthy Internet use	977(34.8)
Low (≤ 300 RMB)	299(10.7)	Accidental injury	1320(47.1)
Medium (300–700 RMB)	1690(60.2)	Tobacco use	194(6.9)
High (≥700 RMB)	816(29.1)	Binge-drinking	327(11.7)
**Father’s education**		Self-injurious behavior	493(17.6)
Below high school	2361(84.2)	Suicide risk	590(21.0)
High school diploma	407(14.5)		
Beyond high school	37(1.3)		

Table 2 displays the results of fitting the latent class models (one to five classes) for the consequence indicators according to the model selection process [25,26]. Given that traditional fit indices may not uniformly point to a single model specification (and may over- or underestimate the number of classes present), we selected a final model specification and the total number of classes by considering issues such as the interpretability of the results, theory and previous findings in the scientific literature. Thus, the 4-class model was selected as the best-fitting model given the totality of the evidence: although the AIC, Pearson *χ*^*2*^, and G^2^ values continued to decrease as the number of classes increased, the 5-class model had a non-significant LMR LRT result and lower classification probabilities than the 4-class model. Finally, the P-values for the LMR test were statistically significant for both the 3- and 4-class models. The lower BIC and *a*BIC values for the 4-class model support our preference for this model.

**Table 2:** Model fit information for competing latent class models, 2015 (N = 2805).

Number of classes	*df*	AIC	BIC	*a*BIC	Pearson*χ*^*2*^	G^2^	LMR LRT *P*-value
**1**	246	22648.50	22696.02	22670.59	5210.64	1151.70	-
**2**	238	21981.96	22082.93	22028.91	588.72	502.39	*P* = 0.000
**3**	229	21829.04	21983.46	21900.85	394.15	331.47	*P* = 0.005
**4**^**a**^	220	21748.09	**21955.97**	**21844.76**	232.71	232.53	***P* = 0.000**
**5**	211	**21747.07**	22008.39	21868.59	**207.57**	**213.50**	*P* = 0.544

*df* = degrees of freedom; AIC = Akaike information criterion; BIC = Bayesian information criterion; *a*BIC = sample-size-adjusted BIC; G^2^ = goodness of fit; (–) not applicable; LMR LRT = Lo–Mendell–Rubin likelihood ratio test. A smaller BIC and AIC indicate a better model fit. A low *P*-value indicates the (K-1)-class model must be rejected in favor of a model with at least K classes.

^a^ Selected as final model.

**Fig 1:**
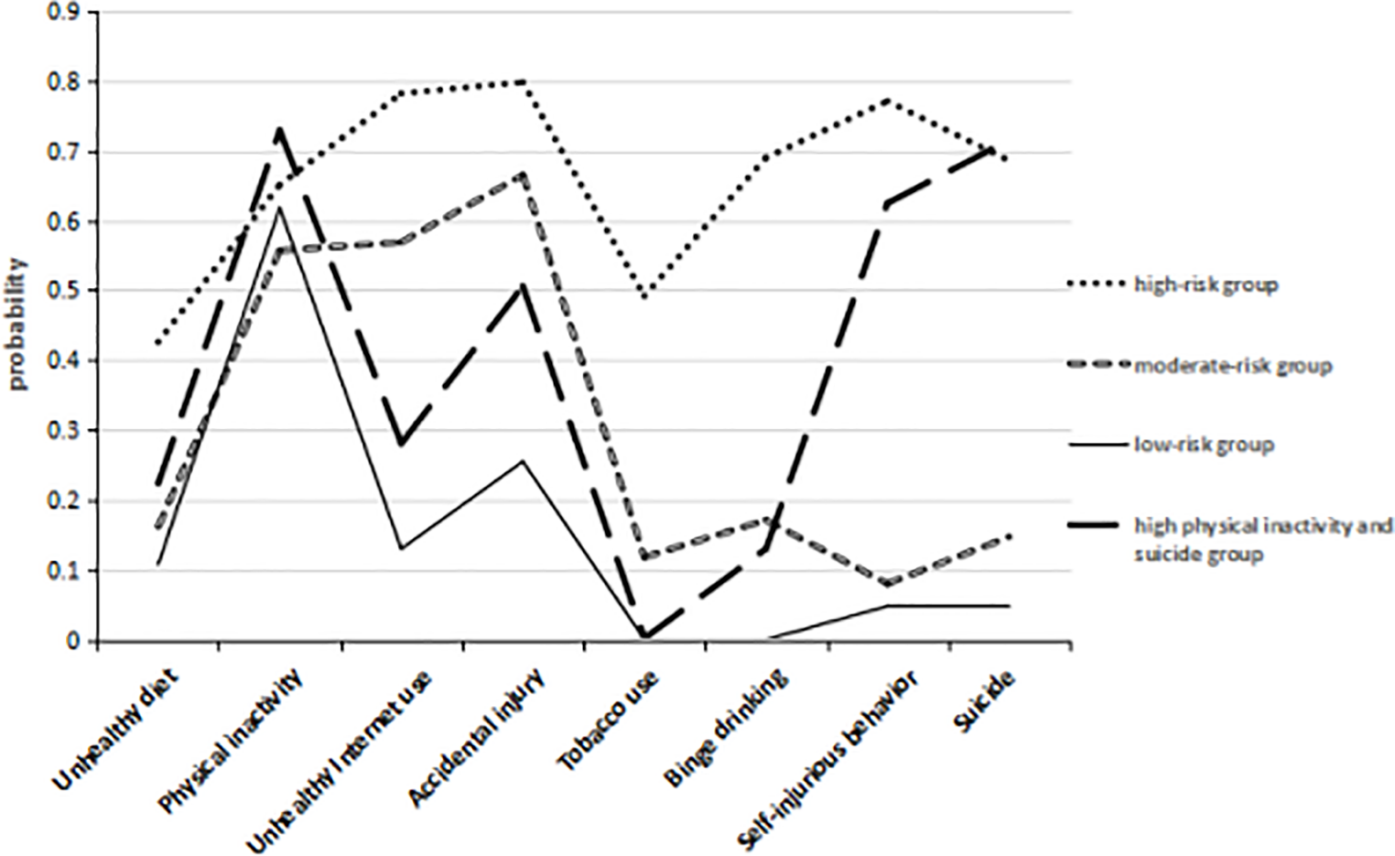
The probabilities of reporting each health risk behavior according to the latent classes.

The probabilities of reporting each health risk behavior according to the latent classes are depicted in Fig 1. We categorized the participating adolescents into the classes with the largest posterior probability and then labeled the classes based on the overall probability of endorsing the consequences and the severity of such consequences. As illustrated in Fig 1, there was no difference between the four classes in the unhealthy diet or physical inactivity behaviors. The high-risk group (n = 108, 4.0%) exhibited the highest probabilities for all eight risk behaviors, and the high-physical-inactivity and suicide-risk groups (n = 340, 12.1%) demonstrated relatively high probabilities of engaging in physical inactivity, self-injurious behavior and suicidal behaviors. Meanwhile, the low-risk group (n = 1460, 52.1%) included the participants who exhibited the lowest probabilities for all risk behaviors. The remaining group (n = 897, 32.0%), called the moderate-risk group, was characterized by relatively high probabilities for unhealthy Internet use, accidental injury and tobacco use.

To examine class differences according to the demographic variables, multinomial logistic regression analyses were conducted. Table 3 presents the results of analyses examining predictors of latent class membership, with “the low-risk group” specified as the reference. Table 3 reveals that (1) boys (odds ratio (OR) = 2.662, 95% confidence interval (CI): 1.753–4.044), participants with poor parental relationships (OR = 4.245, 95% CI: 2.347–7.679), and participants with high monthly allowances (OR = 5.804, 95% CI: 2.142–15.721) were significantly more likely to be in the high-risk group; (2) boys (OR = 3.196, 95% CI: 2.668–3.829), students in grade 10 (OR = 3.196, 95% CI: 2.668–3.829), participants with high monthly allowances (OR = 2.459, 95% CI: 1.714–3.527), participants with poor academic performance (OR = 3.869, 95% CI: 2.042–7.330), and participants with somewhat poor parental relationships (OR = 1.504, 95% CI: 1.183–1.912) were significantly more likely to be in the moderate-risk group; and (3) participants with poor academic performance (OR = 3.625, 95% CI: 1.463–8.983) and those with poor parental relationships (OR = 2.992, 95% CI: 1.973–4.537) were significantly more likely to be in the high-physical-inactivity and suicide-risk group. Notably, being a boy (OR = 0.743, 95% CI: 0.572–0.964) was a protective factor in the high-physical-inactivity and suicide-risk group compared to the low-risk group. Left-behind status, father’s education and mother’s education were not found to be associated with the latent classes of health risk behaviors.

**Table 3:** Multinomial logistic regression results of predicting latent class membership, 2015(N = 2805) ^a^^,^^b^.

Predictors		High-risk group		Moderate-risk group		High-physical-inactivity and suicide-risk group	
		OR	95% CI	OR	CI (95%)	OR	95% CI
**Sex**	**Girls**	-	-	-	-	-	-
	**Boys**	2.662	1.753–4.044	3.196	2.668–3.829	0.743	0.572–0.964
**Grade**	**7th**	-	-	-	-	-	-
	**10th**	1.357	0.751–2.452	1.878	1.457–2.419	1.072	0.777–1.479
**Monthly allowance**	**Low**	-	-	-	-	-	-
	**Medium**	1.783	0.667–4.785	1.203	0.859–1.684	0.974	0.648–1.465
	**High**	5.804	2.142–15.721	2.459	1.714–3.527	1.274	0.803–2.020
**Parental relationship**	**Good**	-	-	-	-	-	-
	**Somewhat poor**	2.161	1.309–3.567	1.504	1.183–1.912	2.266	1.680–3.058
	**Poor**	4.245	2.347–7.679	1.251	0.852–1.838	2.992	1.973–4.537
**Academic performance**	**Good**	-	-	-	-	-	-
	**Moderate**	2.251	0.523–9.686	2.385	1.315–4.328	1.599	0.672–3.805
	**Poor**	4.204	0.922–19.162	3.869	2.042–7.330	3.625	1.463–8.983

OR, odds ratio; CI, confidence interval; (-) not applicable and as the reference.

^a^ Latent class analysis with 4 latent subgroups fit better than the models with 1, 2, 3, and 5 latent subgroups based on fit statistics (i.e., lower values for Akaike information criterion, Bayesian information criterion (BIC), and sample-size-adjusted BIC, higher values for average classification probability, and a low P-value for the Lo–Mendell–Rubin likelihood ratio test, indicating that the 3-class model had to be rejected in favor of a model with at least 4 classes).

^b^ Table 3 presents the results of LCA multinomial regression analyses examining predictors of latent class membership with “the low-risk group” specified as the reference.

## Discussion

Our study results generally support the existing evidence [27–29], suggesting that health risk behaviors are highly interconnected, and they support the notion that the clustering of health risk behaviors may be associated with specific adolescents who are exposed to multiple risk factors (environmental, behavioral), implying their heightened vulnerability [30,31]. Therefore, we identified four classes characterized by unique behavior patterns: a high-risk group (4.0%), a high-physical-inactivity and suicide-risk group (12.1%), a moderate-risk group (32.0%), and a low-risk group (52.1%). The four-class model fit was consistent with the a priori hypothesis and previous studies [20,21,32,33]. However, the proportion of the high-risk group (all health risk behaviors had the highest probabilities) was the lowest compared with the previous studies. For example, Hair et al.’s research [20] revealed four latent classes of adolescents with varying degrees of six kinds of risky behaviors -(smoking, drinking, drug use, no exercise, delinquency, and unsafe sexual behavior): high-risk (26.6%), high levels of drinking and unsafe sexual behaviors (20.8%), low-risk (16.3%), and high levels of smoking, physical inactivity and unsafe sexual behaviors (36.4%). Wang’s study [21] found four latent classes from six kinds of health risk behaviors (suicide/self-injurious behavior, rule breaking, smoking/drinking, unhealthy diet/physical inactivity, aggression/violence and unsafe sexual behavior) of high school students in four cities in China (Shenyang, Changsha, Chengdu and Yinchuan). A high-risk group, representing approximately 13.6% of the sample, had relatively high probabilities of engaging in all types of risky behaviors. Approximately 14.1% of the sample was in a group characterized by very few substance abuse behaviors and a high probability of engaging in suicide and self-injurious behavior. The high-substance-abuse and low-other-risk behavior group represented approximately 27.6% of the overall sample, whereas the low-risk group consisted of those who engaged in very few risky behaviors, representing approximately 44.7% of the overall sample. There are differences between our research and these studies, such as how the groups were labeled and the basis for the classification of adolescent health risk behaviors. These differences may be explained by the following two reasons: 1) the definitions used for health risk behaviors were not completely equivalent. This study did not include drug use and unsafe sexual behavior, as Hair’s research did and did not consider rule breaking and aggression/violence, as did Wang’s research. However, other additional health risk behaviors, such as unhealthy Internet use and accidental injury, were included in our study. 2) The research subjects of the studies were different. The adolescent population of this study came from rural western China, which was very different from Chinese urban areas because of undeveloped economics and unsophisticated folk customs.

Our research revealed that as adolescents’ monthly allowance increased and the quality of their parental relationships decreased, the prevalence of adolescent health risk behaviors increased. Many young parents tend to provide more money to adolescents–whose physical and psychological development is not yet mature–when those parents are unable to remain home and care for their children in rural western China. However, they need spiritual communication more than material goods, especially when they perceive that a poor relationship with their parents is generating a disharmonious family environment. Moreover, our findings are not surprising considering that boys are characterized as risk takers, exhibiting greater levels of engagement in most health risk behaviors and substance use than girls [34]. In the current study, girls with poor academic performance and poor parental relationships were more likely to be in the high-physical-inactivity and suicide-risk group. An earlier study also reported that girls experience higher levels of suicidal thoughts and attempts than boys [35], and our findings support this conclusion and demonstrate the importance of a harmonious family for adolescents’ physical and mental health. Therefore, more attention might be paid to developing a multi-behavioral program that emphasizes the involvement of parents or family members in interventions. Unexpectedly, the left-behind status of participants was not included in the multinomial model. That is, there was no significant difference in the cluster patterns of health risk behaviors between LBC and non-LBC (NLBC). One of the reasons might have been the high proportion (56.9%) of LBC in this study. Left-behind children did not feel that they were a special minority; that is, left-behind status did not bring them psychological pressure, which might indirectly cause problems with health risk behaviors. We found no significant differences in six kinds of health risk behavior between LBC and NLBC, including unhealthy diet, accidental injury, tobacco use, binge-drinking, self-injurious behavior and suicide. Only two health risk behaviors, physical inactivity and unhealthy Internet use, had significantly different prevalence between these two populations. The prevalence (66.5%) of physical inactivity in LBC was higher than that (54.2%) in NLBC, while the prevalence (33.2%) of unhealthy Internet use in LBC was lower than that (37.0%) in NLBC. These results imply that the clustering patterns of the eight health risk behaviors might be similar between LBC and NLBC. Parental education was not a predictor for clustering health risk behavior, which was not consistent with the previous study [5]. Parental college or university degree was an irregular predictive factor for the health risk behaviors of adolescents [5]. The low-level education of parents (the rate of having beyond a high school degree was only 1.3% for fathers and 0.9% for mothers) in our study might limit its influence on health risk behaviors. More research is needed to clarify this relationship.

### Limitations

Our study has several limitations. First, all the participants’ health risk behaviors were self-reported and thus subject to reporting bias. Second, our study was a school-based survey that did not include adolescents who had dropped out of school, and it was conducted only in schools in western rural areas that do not serve urban adolescents. Therefore, our results may not be generalizable to those groups [36]. Third, due to accessibility considerations, we did not use a random sampling method to select the research subjects. Finally, latent class analysis is a type of person-centered approach; thus, different results may be obtained from different samples.

### Conclusions

To our knowledge, this is the first study to utilize LCA to classify different patterns of health risk behaviors among adolescents in rural western China and the first to suggest that rural adolescents are a heterogeneous population requiring different targeted and effective interventions. Our findings have important implications for developing urgently needed and targeted health promotion strategies for adolescents in rural western China. Using empirical methods to identify subgroups and the factors associated with those subgroups has the potential to pinpoint differences in characteristics and may shed light on future directions for tailored and ultimately more effective intervention programs for adolescents in rural western China. Meanwhile, the targeted intervention methods may minimize costs and save health resources.

## Supporting information

S1 Data (SAV)
